# A Mechanistic Review on Protective Effects of Mangosteen and its Xanthones Against Hazardous Materials and Toxins

**DOI:** 10.2174/1570159X22666240212142655

**Published:** 2024-03-14

**Authors:** Roghayeh Yahyazadeh, Vafa Baradaran Rahimi, Ahmad Yahyazadeh, Vahid Reza Askari

**Affiliations:** 1 Department of Pharmacodynamics and Toxicology, School of Pharmacy, Mashhad University of Medical Sciences, Mashhad, Iran;; 2 Department of Cardiovascular Diseases, Faculty of Medicine, Mashhad University of Medical Sciences, Mashhad, Iran;; 3 Department of Histology and Embryology, Faculty of Medicine, Karabuk University, Karabuk, Turkey;; 4 International UNESCO Center for Health-Related Basic Sciences and Human Nutrition, Mashhad University of Medical Sciences, Mashhad, Iran;; 5 Pharmacological Research Center of Medicinal Plants, Mashhad University of Medical Sciences, Mashhad, Iran

**Keywords:** Neuroprotection, neuroinflammation, fruit, xanthones, mangosteen, AMP-activated protein kinases, Parkinson's disease

## Abstract

Due to its pharmacological properties, α-Mangostin, mainly found in *Garcinia mangostana* (*G. mangostana*) L. (Mangosteen, queen of fruits), treats wounds, skin infections, and many other disorders. In fact, α-Mangostin and other xanthonoid, including β-Mangostin and γ-Mangostin, are found in *G. mangostana*, which have various advantages, namely neuroprotective, anti-proliferative, antinociceptive, antioxidant, pro-apoptotic, anti-obesity, anti-inflammatory, and hypoglycemic through multiple signaling mechanisms, for instance, extracellular signal-regulated kinase1/2 (ERK 1/2), mitogen-activated Protein kinase (MAPK), nuclear factor-kappa B (NF-kB), transforming growth factor beta1 (TGF-β1) and AMP-activated protein kinase (AMPK). This review presents comprehensive information on Mangosteen's pharmacological and antitoxic aspects and its xanthones against various natural and chemical toxins. Because of the insufficient clinical study, we hope the current research can benefit from performing clinical and preclinical studies against different toxic agents.

## 1 INTRODUCTION

Nowadays, most chemicals and medicines have exhibited undesirable symptoms and the emergence of drug-resistant pathogenic bacterium, viruses, or fungi, toxic adverse effects of these chemicals, and withdrawal problems, limiting their administration in several countries [1-3]. Hence, the herbal plants' study has produced present medicine with beneficial constituents that have been used to treat various diseases, especially in Africa and Asia [4-7]. Also, they are easily accessible and cheap. Furthermore, many plant species have shown pharmaceutical activities because of the presence of many bioactive ingredients, for instance, flavonoids, alkaloids, steroids, glycosides, saponins, tannins, and terpenes [6-11]. Therefore, herbal medicines have been considered an essential source for finding new pharmaceutical molecules to treat serious diseases [3, 9, 12]. Moreover, some research has proven that flavonoid and phenolic materials display anticancer, anti-inflammatory, antioxidant, and anti-diabetic activities [6, 7, 11, 13, 14].

**Fig. (1):**
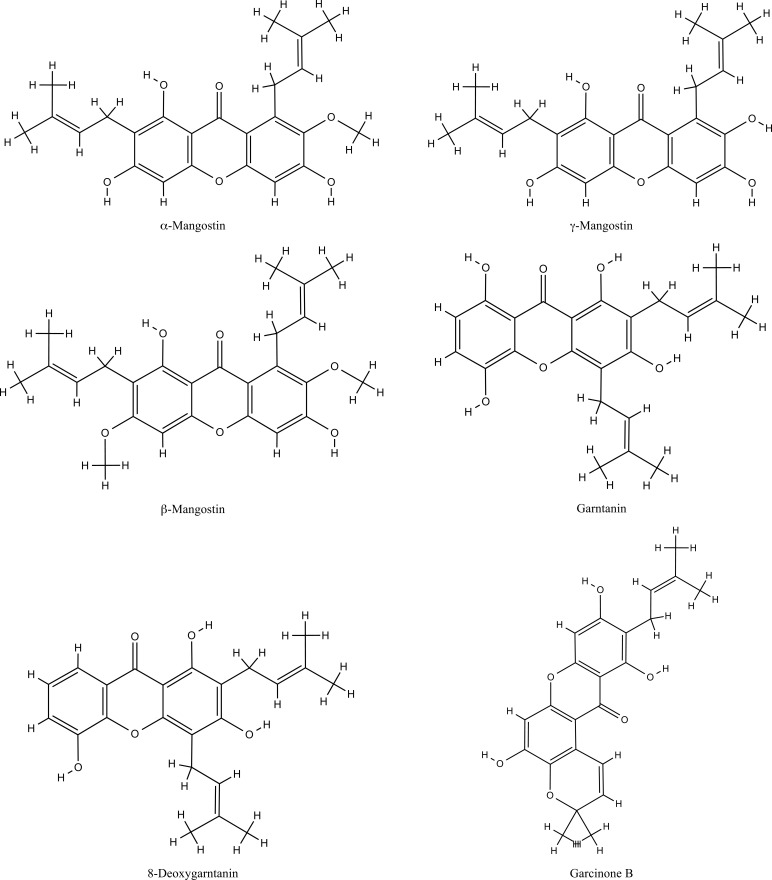
The chemical structure of the main xanthones of Mangosteen.

*G. mangostana* L. (Mangosteen) is an evergreen tree and shrub native to the Philippines, India, Myanmar, Sri Lanka, Malaysia, and Thailand that belongs to the Clusiaceae family. This pyramidal crown tree can reach 6-25 m in height with glabrous, leathery leaves [15]. The color of its fruit is dark purple or reddish, which has white, soft, and juicy edible pulp with acid and sweet flavor [16]. The Mangosteen-driven products classified 12^th^ in the United States upper-selling supplements food products enriched with Mangosteen fruits gained 180 million dollars in the US in 2012 [17]. As queen of fruits, Mangosteen has been used for medicinal purposes by Southeast Asians for centuries in the treatment of skin wounds (that is safe in 8% w/w concentration for skin use) [18] and infections [19, 20], cancer [21], amoebic dysentery, different urinary disorders, cystitis, and gonorrhea [22]. Moreover, the pericarp of Mangosteen-fruit, as a potent antioxidant, is widely used against immunological diseases like arthritis, food allergies, and acne [23] that may modulate the tumor necrosis factor (TNF)-α, interleukin (IL)-1β, IL-6, cyclo-oxygenase-1 (COX-1) and cyclo-oxygenase-2 (COX-2) expressions levels [17]. Some research proved that Mangosteen has myocardial protective [24], neuroprotective [25], and hepatoprotective [26] effects in *in vitro* and *in vivo* investigations. Indeed, prenylated xanthones and dibenzo-γ-pyrone derivatives are the chief complexes in the Mangosteen fruit (Fig. **1**) [27], which possess antioxidant, antibacterial, cytotoxic, and anti-proliferative activity in the *in-vitro* study [28]. Structurally, xanthones have been classified in a unique class of polyphenols consisting of a tricyclic scaffold (C6-C3-C6), which is improved by hydroxyl, isoprene, and methoxyl groups in addition to the A and B rings [29]. Although their biological properties relate to the tricyclic scaffold, various bioactivities are associated with the nature or position of the several functional groups [30-32]. Not only do fruit and pericarp contain xanthones, but also the leaves and bark of Mangosteen are riche of xanthones. Moreover, the research indicated that Mangosteen fruit has notable biological activity [33]. In addition, the secondary metabolite of Mangosteen separately is classified in Table **1**.

In this review, we have emphasized updating and comprehensive report on the toxicological investigations of *G. mangostana* extract and its ingredients on cell-based and animal models with neurological, inflammatory, and metabolic disorders and other toxicities, examining the molecular pathways suggested, and the biochemical factors described to give details about the effects observed and discussed. Taken together, these investigations indicate that Mangosteen and its bioactive xanthones may decline the harmful consequence of chemical and natural toxins and could act as a potential reagent for the treatment of various disorders such as Parkinson’s disease, memory impairment, diabetes, hepatotoxicity, renal dysfunction, and cardiac injury. However, it is necessary to mention that differences in the efficacy of xanthone treatment may differ because of various route administration, dosages, and other dietary components.

The data is gathered from journals that published articles without publication time limitation until the end of December 2022. The keywords used were “protective, or ameliorative,” “toxin, toxic, toxicity, nephrotoxic, radiation, cardiotoxic, hepatotoxic or neurotoxic” with “Mangosteen, Mangostin, Mangostana”. We search through different websites such as Google Scholar (n = 110), PubMed (n = 126), and Web of Science (n = 9). Articles were written in English language and selected according to inclusion and exclusion.

A toxin, as a harmful substance, is generated in living cells or organisms. Natural toxins are often identified from other chemical factors because of their biological roots [34]. Many types of research proved that Mangosteen has a beneficial effect on many natural toxins such as poxvirus, *Staphylococcus aureus,* and various natural toxins (Table **2**).

## 2 ANTIBACTERIAL EFFECTS OF MANGOSTEEN AND ITS INGREDIENTS

Recently, the new antimicrobials' scarceness, as well as elevating antibiotic resistance, has been a severe challenge in the health system, making the demand for practical, affordable, and novel medicines for microbial infection treatment, mostly in developing countries [35, 36]. Some herbal medicines are highly efficient in bacterial infection therapies. Mainly, Mangostin has a magical potential to produce impressive secondary metabolites by elevating the related cytokines [37].

Mangosteen xanthones exhibit that γ-Mangostin, α-Mangostin, and β-Mangostin, as the main derivative substances, have potent antibacterial activities against gram-positive and negative bacteria. For example, γ-Mangostin (with 4.68 µg/ml) showed antibacterial activity against *Staphylococcus aureus* (*S. aureus*). However, α-Mangostin (2.34 μg/mL) displayed more potent activity compared to β-Mangostin (4.68, 2.34, and 9.37 μg/mL) γ-Mangostin (4.68, 18.75, 18.75 μg/mL, respectively) against *S. aureus*, *Bacillus subtilis* (*B. subtilis*), and *Pseudomonas aeruginosa* (*P. aeruginosa*). In this regard, it might be related to the structure of the compounds. Based on the literature review, the methoxy group at C-7 and tetra-oxygenated e skeleton have a crucial role in the effectiveness of these compounds [38, 39]. In addition, γ-Mangostin is more water-soluble because of its high polarity, which reduces lipophilicity and impairs bacterial uptake. Therefore, both adequate water solubility and enough lipophilicity properties improved bacterial uptake and enhanced the antibacterial effect compared to γ-Mangostin [40].

Hydroxyapatite (Hap) has a calcium phosphate structure with biomedical applications in dentistry and orthopedics. In some cases, *Staphylococcus* and *Enterobacter* cause infection in postoperative orthopedic surgeries after the implantation of porous Hap and slow down the improvement of wounds [41, 42]. Therefore, to control debridement, remove the implants, and inhibit this microorganism, Chaiarwut *et al.* surveyed Mangostin-coated Hap granules to prevent bacteria (*Escherichia coli*, *Acinetobacter baumannii*, and *Staphylococcus aureus*) persistence in chronic or long-term infections [41]. Mangostin-coated Hap granules in a dose-dependent manner (0.05 and 0.1 mg/mL of Mangosteen extract) decreased the growth of bacteria during 24 h, reaching 99.99% bacterial diminution without any toxicity against calvaria-derived pre-osteoblastic (MC3T3-E1) cells and calcium deposition [41]. Another study revealed that Mangosteen extract exhibits anti-bacterial activities, and its nano-forming compounds (α-Mangostin-AgNPs, Mangosteen-AgNPs) inhibit the gram-positive and gram-negative bacterial strains. The evaluation of 2, 2-diphenyl-1-picrylhydrazyl (DPPH), 2′-azino-bis-3-ethyl benzothiazoline-6-sulphonic acid (ABTS) free radicals, as an antioxidant activities evaluation manner, showed that Mangosteen-AgNPs and α-Mangostin-AgNPs were able to inhibit the ABTS and DPPH free radical with IC_50_ value of 56.3 and 36.5 μg/mL. In addition, its effectiveness on gram-positive strains' growth inhibition was more than gram-negative strains' growth inhibition, with the least inhibitory effect against *P. aeruginosa* and *E. coli* [42]. Furthermore, it has been proved that the ABTS radical scavenging ability remarkably relates to the number of hydroxyl groups, regardless of their location in the phenolic compounds [43].

Therefore, all research proved that Mangosteen extract and its ingredients have anti-bacterial activities, such as *α*-Mangostin glycosides, which are soluble in water [44]. The investigation revealed that *α*-Mangostin glycosides (50 μg/disc) inhibited *Micrococcus luteus* (*M. luteus*), *B. subtilis*, and *S. aureus* growth. In addition, gram-negative bacterial strains in this study were resistant due to their outer lipopolysaccharide layer thickness. Therefore, the α-Mangostin glycopyranosides could band to a specific site on the bacteria's cell wall to stimulate the antibacterial effects [45].

**Fig. (2):**
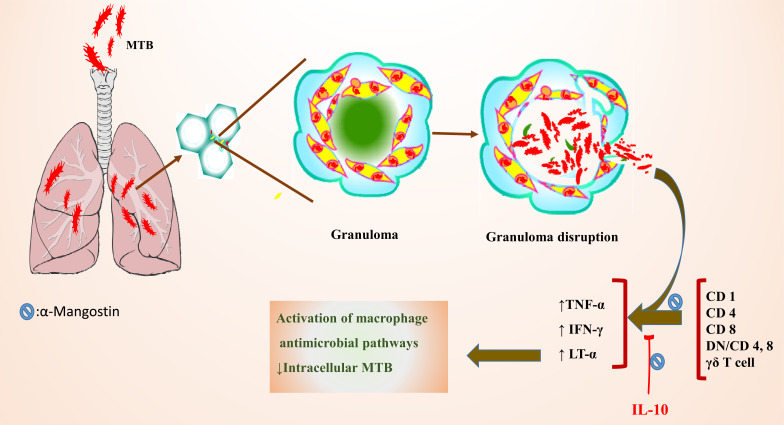
Mechanism and effects of α-Mangostin on MTB infection.

Tuberculosis (TB), a deadly human disease, has been considered a serious threat to humans worldwide in all age groups caused by *Mycobacterium tuberculosis* [46]. Interaction between macrophages and host T-cells affects *Mycobacterium tuberculosis* (MTB) progression, adjusted by cytokines [47]. In active TB individuals, interferon-gamma (IFN-γ) is generated by activating natural killer cells (NK cells), CD8^+^T-cells, and CD4^+^ T-cells. As a result, IFN-γ actives the macrophages and elevates the elimination of intracellular MTB [48, 49]. However, Il-10 has a suppression effect on immunogenicity against TB by reducing the IFN-γ expression level [50] (Fig. **2**). Generally, balance among pro- and anti-inflammatory cytokines adjusts the severity of pathological damage that is relayed to the rate of bacteria in granuloma [51]. Thus, producing new materials is necessary to prevent and treat this disease. Therefore, an *in vivo* study on infected mice (with MTB multidrug-resistant) revealed that the increased IL-10 levels were reduced by 6.95 mg/kg of α-Mangostin for seven days. Moreover, it can increase the IFN-γ expression. In addition, its treatment with α-Mangostin can decrease the severity and colonies of bacteria. Therefore, Mangostin xanthan has adjuvant therapy and immunomodulatory effects on TB [52].

## 3 MANGOSTEEN AND ITS INGREDIENTS' EFFECTS ON LIPOPOLYSACCHARIDE-INDUCED TOXICITIES

Lipopolysaccharide (LPS) is a component of the cell wall of gram-negative bacteria that triggers the initiated signaling cascade for the expression of cytokines (TNF-α, IL-6, iNOS, and NF-κB) as well as cyclooxygenase 2 (COX2) LPS up-regulates prostaglandin E_2_ (PGE_2_) and nitric oxide (NO) [53]. Also, the induced oxidative stress biomarkers *via* nitrogen (RNS) or reactive oxygen species (ROS) have a prominent role in the pathogenesis stimulated by LPS [54]. Hence, the antioxidant effect of Mangostins against LPS-induced oxidative stress has been studied in many *in vitro* and *in vivo* studies that are evaluated in Table **3** [55].

**Fig. (3):**
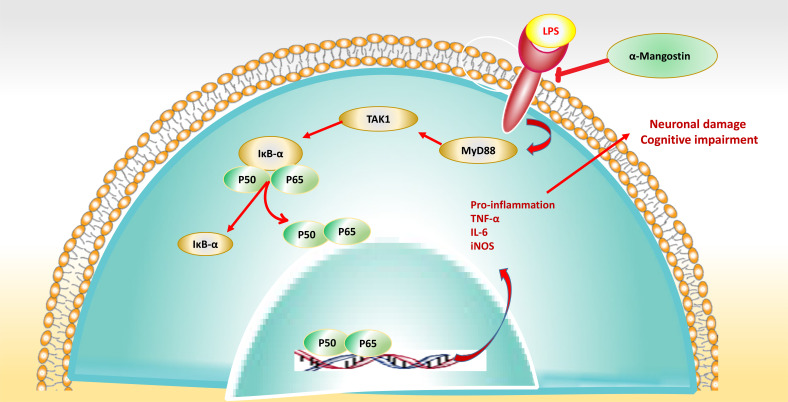
The effect of α-Mangostin as a main xanthone of Mangosteen was evaluated on LPS-induced memory impairment and microglial dysfunction.

In dentistry, bone healing notably depends on the early inflammation stage with systemic and local responses against nasty stimuli. The previous study on α-Mangostin suggested that it was able to improve osteoblastic differentiation against LPS administration. Therefore, the up-regulated IL-1α (in LPS-induced toxicity cells) was decreased *via* 5 μg/ml of α-Mangostin. However, the suppressed ALP in LPS-induced toxicity in osteoblast cells was increased by α-Mangostin [56]. Another investigation revealed that α-Mangostin elevates the Human nucleus pulpous cells (NPCS) viability infected by LPS. In addition, α-Mangostin down-regulated the expression levels of NLRP3 inflammasome (NOD-, LRR- and pyrin domain-containing protein 3), ASC (the modifier molecule apoptosis-depended speck-like protein including a CARD), pro-caspase-1, IL-18 as well as IL-1β. Moreover, treatment with α-Mangostin notably suppressed apoptotic cell death, p-p65/p65, and nuclear p65 levels in NPCs compared to treatment with the LPS group. Also, it not only decreased the Bax and caspase-3 expression but also increased the level of Bcl-2 expression in α-Mangostin-treated NPCs. It is necessary to mention that NF-κB inhibition *via* pyrrolidine dithiocarbamate (PDTC) boosted the suppressed effects of α-Mangostin on apoptosis as well as activation of NLRP3 inflammasome in NPCs. So, α-Mangostin protects NLRP3 inflammasome-assessed apoptosis in LPS-induced NPCs *via* NF-κB signal adjusting (Fig. **3**) [57]. Tianhua Fu *et al.* investigation proved that α-Mangostin (12.5 and 25 mg/kg) has a hepatoprotective effect on LPS-induced acute liver failure in mice *via* the reduction in MDA, TNF-α, aspartate transaminase (AST), serum alanine aminotransferase (ALT), interleukin-1β, -6 levels. In contrast, it can improve hepatic glutathione (GSH), catalase (CAT), superoxide dismutase, and (SOD) activities. In addition, it could down-regulate NF-κB and toll-like receptor 4 (TLR4) expression. In contrast, α-Mangostin stimulated the heme oxygenase-1 (HO-1) and nuclear factor erythroid 2-related factor 2 (Nrf2) expression levels. Moreover, α-Mangostin has hepatoprotective effects on LPS-induced liver dysfunction *via* stimulating the Nrf2 (for antioxidant induction defense) and suppressing the TLR4 signaling pathway(for producing anti-inflammatory effects) [58].

## 4 ANTIVIRAL EFFECTS OF MANGOSTEEN AND ITS INGREDIENTS Α-MANGOSTIN

Investigations showed that the antiviral functions of Mangosteen are related to the polyphenolic compounds (xanthones), such as α-Mangostin and γ-Mangostin, *via* various mechanisms of action, such as inhibiting the reverse transcriptase and interfering with other replication processes [23, 59]. In fact, the section aims to review data on the antiviral activities of Mangosteen and its ingredients for managing viral diseases (Table **3**).

The evaluation of the Mangosteen effect on avian pox virus (APV) revealed that ovo incubation of Mangosteen (1.5% and 3% w/v at 0.1 mL/egg) represses APV *via* decreasing pock lesions on the chorioallantoic membrane of the embryonated chicken eggs [60]. Moreover, an *in-silico* study suggested that the α-Mangostin notably inhibits the main protease enzyme in HIV-1 (human immunodeficiency virus-1) [61-63] and COVID-19 (The main therapeutic target of COVID-19) [64]. Furthermore, the *in vitro* cell assay investigation of α-Mangostin (8 µM under the co-treatment condition) inhibited the dengue virus growth. Then *in-silico* study for detecting the inhibiting mechanism exhibited that α-Mangostin can stick with multiple dengue virus protein objects (glycoprotein E, NS5 methyl-transferase, and NS2B-NS3 protease). Therefore, the results indicated that it could inhibit the production of the dengue virus during the replication cycle. Also, they introduced it to use as a prophylactic and therapeutic agent [65]. Another designed assay proved that α-Mangostin inhibits the rotavirus replication after 1-2 hours but cannot detect the activated antiviral signaling pathways from the compounds that block the virus replication cycle. In addition, it stimulates NF-κB activation and IL-8 expression. Hence, it can reduce rotavirus infectivity [66]. As a result, Mangosteen and its ingredients have beneficial and curative properties on viral diseases by different mechanisms of action, including blocking the viruses' replication cycle, virus growth, decreasing pock lesions, inhibiting the main protease enzyme, and priming the immune system against viruses.

## 5 ANTI-PARASITE EFFECTS OF MANGOSTEEN AND ITS INGREDIENTS Α-MANGOSTIN

The parasite is an organism that lives in or on the host for feeding. Human parasites are categorized into protozoans, helminths, and ectoparasites. They are usually transferred to their hosts by ingesting infected water/food or biting an arthropod as an intermediate host or a vector [67]. This section evaluates some of them and their interaction with Mangosteen and its ingredients (Table **4**).

*Opisthorchis viverrini* (*O. viverrini*) infection is produced by metacercariae of *O. viverrini* in raw cyprinid fish. After ingestion, the liver fluke movement to the biliary tract causes several hepatobiliary disorders such as cholangiocarcinoma (CCA), cholangitis, lithiasis, and hepatitis [68]. The investigation of α-Mangostin on *O. viverrini* revealed that α-Mangostin (142.04 μg/ mL) has antioxidant activities. Furthermore, histopathological evaluation of the hepatobiliary system showed that α-Mangostin admiration for 45 days could suppress the inflammatory cells surrounding the hepatic bile duct. In addition, in the adult *O. viverrini*, the body size was smaller, and egg production was reduced in Mangosteen-treated hamsters compared to the control group [69].

*Nilaparvato lugens* Stat., brown planthopper (BPH), is a common devastating insect pest in rice farms and can cause the loss of rice crops. Topical spraying of *G. mangostana* L. (with LC_50_ of 4.5% w/v) and α-Mangostin (LC_50_ of 5.44% w/v) as an alternative control agent against BPH Thailand strain, which affects various stages of nymphal and adult BPH. It has been shown that the extract and its active constituent α-Mangostin spraying had no dermal irritation and intoxication but exhibited eye irritation for one day (Table **4**). In fact, glutathione-s-transferase (GST), carboxylesterase, and acetylcholinesterase are the main detoxification enzymes observed after 24 hours of exposure. On the other hand, toxicity values (LC_50_) of Mangosteen extract elevated in each generation. Moreover, The LC_50_ rates for each generation were estimated at 4.22-6.67 after continuously spraying. Therefore, Mangosteen pericarp extract is an alternative insecticide against BPH, which is effective without environmental pollution and resistant to BPH [70, 71].

Malaria, as one of the parasitic tropical diseases, is routinely treated with aminoquinolines, antifolates, artemisinin derivatives, and the hydroxyl naphthoquinone atovaquone [72]. The mechanism of this parasite is described in Fig. (**4**) [73]. Unfortunately, the adverse effects of these compounds and the economic burden motivated the researchers to investigate other alternatives or sensitizers for medications. The antimalarial mechanism of Mangosteen is not clear yet, but its hydros show potent anti-plasmodial activities by inhibiting hemozoin production. Hemozoin is produced from blood digestion by some blood-feeding parasites like plasmodium. Because of free heme toxicity on parasites, it is converted to an insoluble crystalline called hemozoin and is known as a malaria pigment [74, 75]. The *in vitro* investigation of Mangosteen extract was performed against chloroquine-sensitive (strain 3D7) and chloroquine-resistant (strain K1) *Plasmodium falciparum* (*P. falciparum*). Median (range) IC_50_ values of the α-Mangostin and 9-hydroxy calabae for strain 3D7 *vs.* strain K1 clones were 17.9 (15.7.0-20.0) *vs.* 9.7 (6.0-14.0) μM, 1.5 (0.9-2.1) *vs.* 1.2 (1.1-1.6) μM, respectively. Analysis and combination index of α-Mangostin with 9-hydroxy caliber combination showed the synergistic antimalarial activity in both clones 3D7 and K1 strains [76]. Another research showed that Mangosteen had a weak and moderate anti-plasmodial activity with inhibiting concentration (IC50) (range) values of 11.12 (10.94-11.29) and 7.54 (6.80-7.68) µg/ml. Nevertheless, in this study, the Median (range) parasite density in the mice treated with 250, 500, 1000, and 2000 mg/kg of the Mangosteen extract for 14 d was 11.4 (9.49-13.8), 11.6 (9.9-12.5), 11.7 (10.6-12.8), 10.9 (9.4-11.6) that is very low compared to the control group. Therefore, Mangosteen had weak antimalarial properties in this study, which may be due to insufficient absorption of the active complexes [77].

*Caenorhabditis elegans* (*C. elegans*), as a multicellular organism model for basic medical and biological study, is a kind of nematode that is used to test a potential anti-parasitic effect of α-Mangostin. The investigation indicated that α-Mangostin decreased the growth of the *C. elegans* flock (LC_50_: 3.8 ± 0.5 μm), similar to mebendazole effects [78]. Therefore, according to the mentioned contents, Mangosteen and its xanthones can be used as an alternative therapy against various parasites.

## 6 EFFECTS OF MANGOSTEEN AND ITS INGREDIENTS AGAINST FUNGAL TOXICITIES

*Candida albicans* (*C. albicans*) growth in dentures, as a yeast biofilm, is a common disease in dentistry. Therefore, the *in vitro* investigation of α-Mangostin (concentration of ≥ 2,000 μg/ml) decreased the germ tube formation and the *C. albicans* adhesion to denture compared with clotrimazole (a topical anti-fungal treatment) that was similar [79]. In some studies, the efficacy of α-Mangostin may happen through the destruction of mitochondrial energy metabolism [80]. *Aphanomyces invadans* (*A. invadans*), *Achlya bisexualis* (*A. bisexualis*), and *Saprolegnia diclina* H3 (*S. diclina* H3), as aquatic fungi, belong to the *Saprolegniaceae* family that could make infection the fish or eggs like Saprolegniasis [81]. α-Mangostin with 125, 250 ppm for *A. invadans*, 250 ppm for *A. bisexualis*, and 125 ppm for *S. diclina* H3 could cause malformed growth of hypha, with the dense and short branches in the inhibition zone of mycelium. Also, α-Mangostin (500, 1000 ppm) could inhibit the zoospores' growth and had toxic effects on them [82]. Therefore, the low toxicity, rapid and robust properties of the anti-fungal properties of Mangosteen and its ingredients make it an alternative candidate treatment. However, the results of *in vivo* and *in vitro* studies' significance must be verified by clinical research and must be investigated.

## 7 EFFECTS OF MANGOSTEEN AND ITS INGREDIENTS AGAINST ARTHROPODS

As an important pest to most agricultural products, the rice weevil *Sitophilus oryzae* L. destroys about 70% of agricultural crabs yearly [83]. After a whole drilling into a seed or grain kernel, the female puts in a single egg and seals. The development of larva and pupation is performed in the seed or grain kernel. At last, the seed or grain is left after 2-4 days. Therefore, it damages the rice or grain [84, 85]. The *in vitro* study revealed that the peel of Mangosteen extracts with LC_50_: 4.91 ± 1.19% w/v for 12 hours down-regulated the esterase and GST expressions in lived rice weevil [86]. Also, another investigation revealed that Mangosteen extract (LC_50_: 30.1 µg/ml) and α-Mangostin (LC_50_: 19.4 µg/ml) had substantial toxicity against the larvae of *Aedes aegypti* [87]. The results suggest that Mangosteen and related ingredients should be utilized for their potential property for controlling insects/arthropods and associated diseases.

## 8 EFFECTS OF MANGOSTEEN AND ITS INGREDIENTS AGAINST IONIZING RADIATION

Exposure to ionizing radiation (IR) triggers the complex systemic cascade as well as tissue-specific reactions that produce damaging signal transduction, impair the cell's functions, and produce free radicals [88]. ROS or reactive nitrate species (RNS) stimulate NF-κB p65 translocation in the nucleus by phosphorylation of IκBα [89]. So, they lead to the overproduction of IL-1β, IL-6, TNF-α and TGF-β activation [90]. Administration of Mangosteen extract (500 mg/kg) for 30 days notably reduced the imbalance state of redox and toxicity motivated by γ-rays in liver tissues. Furthermore, it downregulates the ameliorated TGF-β1, MDA, NO, ALT, AST, ALP expressions, and NF-κB transcriptional factor and upregulates the SOD and CAT expressions. Mangosteen also had a suppressing effect on biomarkers such as CRP, TNF-α, and IL-6. These alterations make a proliferating improvement in cell nuclear antigen, which is expressed in the cell nuclei and is essential for DNA repair/synthesis [91]. As a result, Mangosteen could improve oxidative damage, pro-apoptotic alternations, and inflammations induced by IR.

## 9 MANGOSTIN AND ITS CONSTITUENTS' EFFECTS ON H_2_O_2_-INDUCED TOXICITY

Mangosteen and its ingredients have many beneficial effects against many chemical agents, including pesticides, chemicals, and heavy metals, as explained below (Tables **5** and **6**).

H_2_O_2_ induces cell death in biotic and abiotic stress and causes ROS generation [92, 93]. ROS directly affects mitochondrial morphology and triggers programmed cell death [94]. Moreover, H_2_O_2_ can change the VEGF expression and its receptors, as well as the transcription factor of NF-κB in endothelial cells [95]. The investigation revealed that hydroethanolic Mangosteen extract (320 μg/mL) for up to four hours could protect and improve DNA damage of H_2_O_2_-induced oxidative stress in human leukocytes [96]. In another experiment, α-Mangostin (10 and 30 µM) potentially inhibited lipid peroxidation (LPOs) and oxyhemoglobin oxidation pathways and enhanced the cell-protective ability on H_2_O_2_ -induced acute oxidative stress in the erythrocytes [97]. The study by Jittiporn *et al.* revealed that Mangosteen (1, 5, and 10 μg/ml) inhibited H_2_O_2_-induced ROS formation and cell death in human endothelial cells (Table **5**). Also, it attenuated p38 MAPK, poly (ADP-ribose) polymerase-1, and caspase 3 cleavage [98]. Briefly, all investigation demonstrates that Mangosteen and its ingredients have anti-apoptotic effects on H_2_O_2_-triggered cell death by preventing ROS formation. Many studies explained the antitoxic effects of Mangosteen and its xanthones against various toxic compounds in Table **5**.

## 10 MANGOSTEEN AND THE EFFECTS OF ITS INGREDIENTS ON DRUG TOXICITIES

### 10.1 Cisplatin

**Fig. (5):**
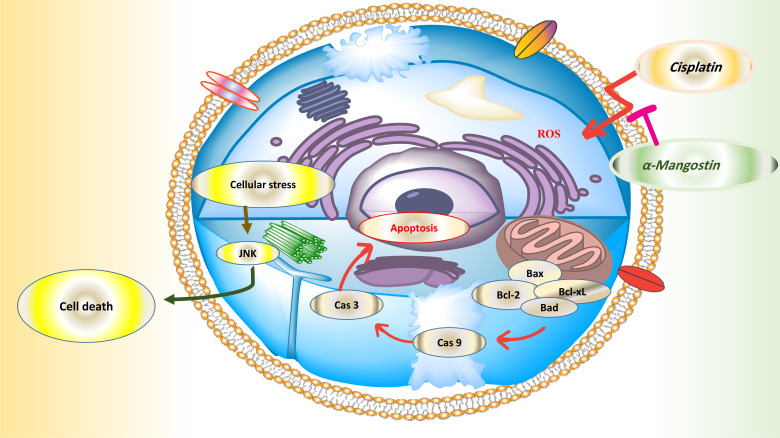
Evaluation of α-Mangostin against cisplatin-induced toxicities.

As a known anti-tumor activity, cisplatin triggers several signal transduction pathways for inducing apoptosis *via* interaction with DNA to create the DNA adducts [99, 100]. Many reasons are reported for cisplatin resistance, such as elevating inactivation *via* thiol-molecules, repair of damaged DNA/its tolerance, creation of intracellular accumulation, upregulating of apoptosis inhibitors (un-adjusted cell signaling pathways, and surviving) (Fig. **5**) [99]. In addition, cytoprotective compounds such as α-Mangostin may diminish its adverse effects (nephrotoxicity, neurotoxicity, and ototoxicity). α-Mangostin (2.5 µg/ml for 16 h) remarkably decreased the cisplatin-induced cytotoxicity and enhanced the apoptotic effects (Table **6**). Whereas α-Mangostin at a concentration higher than 5 µg/ml did not show any cytotoxicity effect but inhibited 100% cell growth [101]. It is worth mentioning that α-Mangostin is capable of enhancing the cytotoxic effects of cisplatin on cancer stem cells and tumor growth [102, 103] through induction of mitochondrial apoptosis signaling and depolarization (over-expression of Bax, reduction of Bcl-2, Mcl-1 and caspase-9/3 activation) [104].

### 10.2 Streptozotocin

Streptozotocin (STZ), an approved drug by the Food and Drug Administration (FDA), was suggested for the therapeutic of metastatic cancer of the pancreatic islet cells. Because of its adverse effects, its usage was limited to patients whose disease could not be improved by surgery. STZ decreased the tumor size and hypoglycemia through the over-secretion of insulin [105]. In the experimental animal study, STZ was used for inducing insulitis and diabetes (because of its high toxicity to beta cells) [106] and Alzheimer's disease [107]. STZ, similar to glucose, causes transportation into the cell by the glucose transport protein GLUT2, which makes it toxic to beta cells as these kinds of cells have a large number of GLUT (Table **6**). However, the other transport proteins are unable to transport the STZ [108, 109]. γ-Mangostin, as a pigment of *G. mangostana*, presents hydrogen atoms for neutralizing free radicals. Therefore, it can decrease oxidative stress, especially in damaged cells, because of its prolonged hyperglycemic states in renal proximal tubular cells and hepatocytes [110-112]. The *in vivo* study revealed that administering γ-Mangostin in dosages of 1, 2 and 4 mg/kg reduces the creatinine and plasma BUN and can also curate the damaged renal proximal tubular cells in STZ-induced diabetes mice [113]. The investigation of α-Mangostin on STZ-induced diabetes mice showed that α-Mangostin at doses of 2, 4, and 8 mg/kg decreased plasma creatinine and BUN (Table **6**). Also, it had a curative effect on impaired renal proximal tubular cells of the kidney in diabetic mice [114]. Hence, Mangosteen and its xanthones have been introduced as a high-potent antioxidant agent to prevent diabetes mellitus or clinical management or reduce the STZ-induced adverse effects in patients (Table **6**).

## 11 THE EFFECTS OF MANGOSTEEN AND ITS INGREDIENTS ON INSECTICIDE-INDUCED TOXICITIES

The use of a wide range of chemicals to destroy pests and weeds is needed to achieve better production in agriculture (Table **6**). Nevertheless, this compound's over-use causes widespread concern over its adverse health effects. Currently, pesticides are different in their mechanism of action, uptake, metabolism, elimination from the body, and human/animal toxicity. In addition, the active ingredients create adverse effects and contain impurities, solvents, carriers, emulsifiers, carriers, and other product constituents (Table **6**) [115].

### 11.1 Abamectin

As an insecticide and anthelmintic compound, abamectin is naturally produced by *Streptomyces avermitilis* [116]. The insecticide activity of abamectin is induced by interference in insects’ nervous systems during different developmental stages and stimulates the glutamate-gated chloride channel [117]. Its metabolism occurs by cytochromes P450. Furthermore, the neurotoxicity effects of abamectin are induced by apoptosis and oxidative stress. The administration of α-Mangostin (20 mg/kg) at 6-19 gestational days reduced the developmental neurotoxicity of abamectin *via* lowering the levels of NO and MDA and increasing the level of GSH, GST, SOD, CAT biomarkers, catecholamine’s as well as apoptosis-proteins (with the regulation of the reelin and nestin genes). It is suggested that the neuroprotective efficacy of α-Mangostin occurs *via* transcription regulation of reelin and nestin (Table **6**). Also, it regulated the serotonin and dopamine concentrations in the fetal brain of rats that were induced neurotoxicity *via* abamectin [118].

### 11.2 Rotenone

As an insecticide and pesticide, rotenone belongs to the rotenoids family, which is a colorless, odorless, crystalline isoflavone compound [119]. Recently, rotenone has been widely used to induce Parkinson's disease in rodents, and its mechanism is explained in Fig. (**6**). Also, it interfered with oxidative stress, degeneration of dopaminergic neurons, and α-synuclein (α-Syn) accumulation by suppressing mitochondrial activity [120].

Parkinson’s disease is a neurodegenerative disease in the elderly. Dopamine depletion is the major reason for motor symptoms caused by neuron degeneration in substantia nigra pars compacta *via* apoptosis. The Bcl-2 and caspase families adjust apoptosis through the intrinsic/extrinsic routes. Caspase-9 activation initiates the mitochondria-mediated pathway. Moreover, activation of caspase-8 triggers the cell death receptor-mediated pathway. Both initiator caspases affect executioner caspases (caspase-3 and -6). In turn, the executioner caspases cause apoptosis feature (DNA fragmentation). Proapoptotic factors, such as Bax, as a proapoptotic factor, are essential in dopaminergic neuron death in Parkinson’s disease [121].

The administration of α-Mangostin (40mg/kg) before the rotenone injection for nine weeks potentially improved locomotors and cognitive impairments (Table **6**). α-Mangostin treatment enhanced the reduced autophagic markers (LC3II/I, TFEB, Beclin-1) *via* up-regulating the AMPK pathway in the cortex and striatum. At the same time, it caused a down-regulation of α-Syn expression in the striatum. α-Mangostin also improved the loss of dopaminergic fibers in the striatum [122]. In another study, treatment with 10 mg/kg α-Mangostin, as a polyphenolic xanthone, for 21 days reduced α-Syn accumulation, which in turn preserved against tyrosine hydroxylase positive dopaminergic neuron loss in rats with rotenone-induced Parkinson's diseases [123].

## CONCLUSION AND RECOMMENDATIONS

Mangosteen and related active secondary metabolites xanthones (mainly α-, β- and γ-Mangostin) were identified in only a few plant families, lichens as well as fungi that displayed many pharmacological through various mechanisms with good safety in the major organ systems. Mangosteen, with other chemotherapeutic agents or alone, is an extraordinary therapeutic agent with lower toxicity. Moreover, the Mangosteen-related study displays a promising approach to treating complex diseases (Alzheimer’s disease, Parkinson's disease, hepatotoxicity, and cardiac and kidney dysfunction). In addition, it is capable of reducing/inhibiting the harmful effects of chemical/natural toxic substances by savage the free radical generation. Mangosteen and its ingredients can treat acne and cause the skin to turn light by reducing the melanin concentration. According to the above studies, they can downregulate the cytokines expression such as TNF-α, MDA, SOD, IL-1β, IL-6, cyclo-oxygenase-1 (COX-1), and cyclo-oxygenase-2 (COX-2) expression levels. Therefore, we believe that Mangosteen and its xanthones can be used to treat certain viral and microbial diseases. In addition, these compounds have been studied for their possible effects on the new strain of viruses *via* molecular docking. However, low oral bioavailability and poor water solubility limit their therapeutic usage. This article reviews and discusses detailed information about Mangosteen's phytochemical and pharmacological properties and its ingredients against natural and chemical toxicities. However, excessive Mangosteen usage may have harmful consequences for people's health. However, there has not been any clinical study available that would demonstrate the efficacy of Mangosteen and its major xanthones, including α, β, and γ-Mangostin, in humans-toxicities. Therefore, xanthones or extracts of *G. mangostana* may have remarkable clinical potential against natural and chemical toxicities in humans; however, further *in-vivo* and clinical studies are needed.

## Figures and Tables

**Fig. (4):**
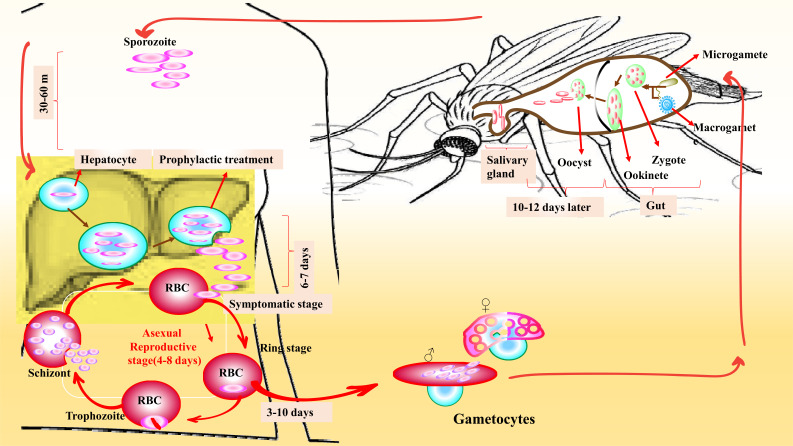
Summarized *Plasmodium* spp. parasites' reproduction mechanism and life cycle in the human host, which is infected *via* an *Anopheles* spp. Mosquito bite.

**Fig. (6):**
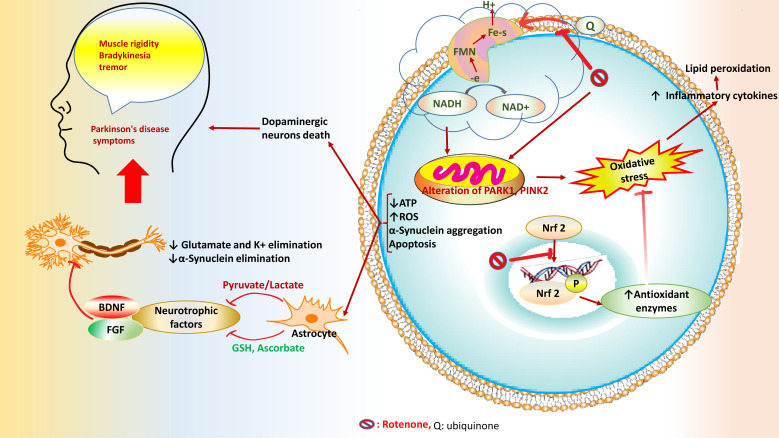
The effect of rotenone on regulating the Nrf2 signaling pathway and induction of Parkinson’s disease was proposed. Rotenone/ROS induces mitochondrial damage and change in mitochondrial proteins (PINK1/Parkin (PARK 2). A-synuclein aggregation and apoptosis are the main prognoses for the onset of Parkinson's disease.

**Table 1:** Classification of secondary metabolites of Mangosteen.

**Part of Plant**	**Type of Compounds**	**Compound Name**
Pericarp	Xanthones	α –Mangostin [33, 124, 125], β –Mangostin [126], γ-Mangostin [127], 1,3,6,7-Tetrahydroxy-2,8(3-methyl-2-butenyl) e P1 [128], 1,3,6-Trihydroxy-7methoxy-2,8-(3-methyl-2butenyl) e P2 [128], 1,3,8-Trihydroxy-4methyl-2,7-diisoprenyle [124], 1,5-Dihydroxy-2-(3methylbut-2-enyl)-3methoxy-e [129], 1,5-dihydroxy-2-isopentyl-3-methoxy e [130], 1,7-Dihydroxy-2-(3methylbut-2-enyl)-3methoxy-e [129, 131], 1,7-dihydroxy-2-isopentyl-3-methoxy e [130], 1-IsoMangostin [16, 130], 1-IsoMangostin hydrate [31], 2-(γ ,γ -Dimethylallyl)-1,7dihydroxy-3-methoxye [19, 31], 3-IsoMangostin [19, 130], 3-IsoMangostin hydrate [19, 130], 8-Deoxygartanin [124, 130], 8-Hydroxycudrae [16], BR-e [31], Cudrae G [16], Garcimangosone B, C [124, 132], Garcinone B, D, E [33, 130], Gartanin [19, 33, 129], Mangostanin [131], Mangostenol, Mangostenone A, B, Mangostinone, [22, 124], Smeathe A [16], Tovophyllin A, B, Trapezifolie [16, 124].
	Benzophenones	Garcimangosone D [132], Maclurin [19, 130], Kolanone [130]
	Flavonoids	Epicatechin [128, 133]
	Anthocyanins	Chrysanthemin [130, 131], Cyanidin-3-O-sophoroside [130].
Fruit	Xanthones	α –Mangostin [33, 124], β –Mangostin [126], γ-Mangostin [134], 1,2-Dihydro-1,8,10trihydroxy-2-(2hydroxypropan-2-yl)-9-(3methylbut-2-enyl)furo[3,2a] xanthone-11-one [135], 1,3,7-Trihydroxy-2,8-di-(3methylbut-2-enyl)-e [135], 11-Hydroxy-1-isoMangostin [33], 5, 9-Dihydroxy-8-methoxy2, 2-dimethyl-7-(3methylbut-2-enyl)-2H, 6Hpyrano-[3,2,6]-xanthone-6-one [87], 6-Deoxy-7demethylmangostanin [135], 8-Deoxygartanin [33, 130], Demethylcalabae [19, 131], Garcimangosone A [124, 132], Garcinone B, C, D, E [33, 130], Gartanin [19, 31, 33], Mangostanol [31, 124], Thwaitesie, Mangostenone C, D, E [33], Mangostinone [22, 33, 129],
Stem	Xanthones	α –Mangostin [124, 136], β –Mangostin [126], 1,6-Dihydroxy-3,7dimethoxy-2-(3-methylbut2-enyl)- [137, 138], Mangosharin [87], Mangostanol [31, 124],
Arils	Xanthones	α –Mangostin [22, 124], 2-(γ ,γ -Dimethylallyl)-1,7dihydroxy-3-methoxye [19, 130], 2,8-bis-(γ,γ-Dimethyallyl)1,3,7-trihydroxye [19, 130], Calabae [19, 130], Demethylcalabae [19, 131]
Seed	Xanthones	α –Mangostin [124, 139], Demethylcalabae [19, 131]
Heartwood	Xanthones	(16E)-1,6-Dihydroxy-8-(3hydroxy-3-methylbut-1enyl)-3,7-dimethoxy-2-(3methylbut-2-enyl)e [124, 138], (16E)-1-Hydroxy-8-(3hydroxy-3-methylbut-1enyl)-3,6,7-trimethoxy-2(3-methylbut-2-enyl)e [124, 138], 1,3,6,7-Tetrahydroxy e [130], 1,3-Dihydroxy-2-(2hydroxy-3-methylbut-3enyl)-6,7-dimethoxy-8-(3methylbut-2-enyl)-e [124, 138], 1,6-Dihydroxy-2-(2hydroxy-3-methylbut-3enyl)-3,7-dimethoxy-8-(3methylbut-2-enyl)-e [124, 138], 1,6-Dihydroxy-3,7dimethoxy-2-(3-methylbut2-enyl)-8-(2-oxo-3methylbut-3-enyl)-e [124, 138], 1,6-Dihydroxy-8-(2hydroxy-3-methylbut-3enyl)-3,7-dimethoxy-2-(3methylbut-2-enyl)-e [124, 137, 138], 1-Hydroxy-2-(2-hydroxy3-methylbut-3-enyl)-3,6,7 trimethoxy-8-(3methylbut-2-enyl) e [124, 138], 1-Hydroxy-8-(2-hydroxy-3-methylbut-3-enyl)-3,6,7-trimethoxy-2-(3-methylbut-2 enyl)-e [124, 138], Garciniafuran [138],
	Benzophenones	Maclurin
Leaves	Xanthones	1,5,8-Trihydroxy-3methoxy-2-(3-methylbut-2enyl) e [130] 1,6-Dihydroxy-3-methoxy2-(3-methyl-2-buthenyl)e [130]

**Table 2:** Effects of Mangosteen extract and its xanthones against natural toxins.

**Toxin**	**Model**	**Constituents**	**Results**	**References**
*P. acnes, S. aureus, * *S. epidermidis*, *S. pyogenes*	*In vivo*	α-Mangostin (0.78, 3.13, 0.78, and 6.25 µg/ml) for 24 h	↑Antibacterial activity	[140]
*P. acnes, S. epidermidis*	*-*	Fruit rinds of Mangostin extract (MIC, MBC: 3.91 g/ml) (MIC: 3.91 µg/ml, MBC: 15.63 µg/ml)	↑ Antibacterial activity	[37]
*S. epidermidis*	*-*	α-Mangostin (MIC: 1.215, MBC: 5 μg/mL) for 5 min	↓Biofilm formation ↓Viable bacterial load and aggregation	[141]
*B. subtilis, C. albicans, E. col, * *P. aeruginosa, S. aureus, * *Mycobacterium Antiprotozoal * *activity (P. falciparum, L. infantum, T. cruzi, T. brucei)*	-	α-Mangostin: IC_50_=3.9 µg/ml IC_50_ >200 µg/ml IC_50_=7.8 µg/ml IC_50_=3.7 µg/ml IC_50_= 2.2, 8, 8.9, 7.9 µM	↑Antibacterial activity ↑Antiprotozoal activity	[142]
*M. tuberculosis*	-	α-Mangostin (MIC: 62 μg/mL) (7 and 6 μg/mL, respectively)	↑Inhibit M. tuberculosis growth and replication ↑Autophagy ↓Intracellular bacteria ↑Immune response	[143]
P. acne	-	α-Mangostin (1 mg/g) for 72	↑Antibacterial activity safe for skin use	[18]
*S. aureus, * *P. acne*	-	(MIC: 3.9 and MBC: 7.8 μg/ml) for 24, 72 h	↑Inhibition the growth and kills the bacteria	[144]
P. acne, *S. aureus*	*-*	Mangostin extract (MIC: 0.039 mg/ml and MBC: 0.156 mg/ml)	↑Inhibition the growth and kills the bacteria	[145]
*S. aureus, B. cereus*	-	α-Mangostin (MIC: 0.78-1.56 μg/mL)	↓Cytoplasmic membrane integrity induced vesicle lysis in bacteria ↑bactericidal action	[146]
*S. aureus*	-	α-Mangostin (1.57-12.5 μg/mL)	↑Inhibition of the growth of the bacteria	[147]
S. mutans	-	Mangostin extract ( MIC: 250 μg/ml and MBC: 1000 μg/ml)	↑Inhibition of the growth and kills the bacteria	[148]
*E. faecalis*	-	α-Mangostin (MIC: 1.97 and MBC: 3.94 μg/ml) for 10 m	↑Inhibition of the growth and kills the bacteria	[149]
*B. subtilis*	-	Mangostin extract, GMC, GME: (MIC: 15.625, MBC: 500 μg/mL)	↑Inhibition of the growth and kills the bacteria	[150]
*B. cereus*	-	GMC: (MIC: 1.9531, MBC: 500 μg/mL) GME: (MIC: 7.8125, MBC: 125 μg/mL)	-	[150]
*B. megatarium*	-	GMC: (MIC: 15.625) GME: (MIC: 15.625, MBC: 500 μg/mL)	-	[150]
*B.pumilus*	-	GMC: (MIC: 15.625) GME: (MIC: 62.5, MBC: 500 μg/mL)	-	[150]
*L. rhamnosus, E. faecalis*	*Ex vivo*	α-Mangostin (10,000 mg/L) for 72 h	↑ Inhibition of the cell growth in all sampling depths	[151]
*S. aureus*	*In vitro*	LS02 (MIC: 1.56-6 HC_50_: 103.4 ± 2.1 μg/mL HC_50_/MIC: 17-66.25 μg/mL) for 30 min	↑High membrane selectivity ↑Destruct the bacterial membrane ↑Bactericidal activity ↓Cell toxicity ↓The bacterial resistance ↓CSH ↓Toxicity toward mammalian cells	[152]
	*In vivo: * mice corneas	LS02 (0.5% v/w) for 3 days		
*P. acnes*	*In vitro*	α-Mangostin (MIC: 1 mg/g)	↑ Inhibition of the growth and killed the bacteria	[153]
*S. aureus*	*In vitro*	α-Mangostin (MIC: 7·8-31·25µg ml^-1^, MBC: 31.25-62.5 µg ml^−1^)	↑ Inhibition of the growth and kills the bacteria	[154]
*F. columnare*	*In vitro*	Mangostin pericarp extraction (IC_50_: 23 mg/L and MIC: 10 mg/L) for 24 h α-Mangostin (MIC: 41.0 mg/L and IC_50_: 12.3 mg/L) for 24 h γ-Mangostin (MIC: 4.0 mg/L and IC_50_: 7.5 mg/L) for 24 h	↑ Inhibition of the growth and kills the bacteria	[40]
*Leptospira*	*-*	γ-Mangostin and garcinone C, and the 2 e analogs, 1,3,8-trihydroxye and 1,3-dihydroxythioe (MIC: 100 to ≥ 800 µg/ml)	↓Growth of leptospira	[155]
*Leptospira *(pathogenic, non-pathogenic)	*-*	Crude extract (MIN: 200 to ≥ 800 μg/ml) Garcinone C, γ-Mangostin (Pathogenic, non-pathogenic MIC: 100, 200 μg/mL)	↓Growth of leptospira	[156]
E. coli, B. subtilis, S. aureus, P. aeruginosa	*-*	Semi-synthesis products of α-Mangostin (50 and 100 μM/mL)	↑Antibacterial activity	[157, 158]
*S. mutans*	*-*	α-Mangostin (150 μM)	↓Insoluble EPS→ ↑disrupts the accumulation and acidogenicity ↑Compromises the mechanical stability ↑Inhibits GtfB and GtfC enzymes activity ↓Acid production and the acid tolerance of S. mutans biofilm cells ↓Disrupts ATPase and PTS activities	[159]
*S. mutans*	*-*	α-Mangostin (12 and 120 μM L^–1^, IC_50_: 20-30 μM L^–1 ^at pH 7.0)	↑Inhibition of acid production by *S. mutans* ↑Inhibition membrane enzymes (F-ATPase) ↑Inhibition of glycolytic enzymes ↑Inhibition of alkali production ↑Inhibition of respiration (NADH oxidase) ↑Killing the bacteria	[160]
*S. aureus*	*-*	Nano α-Mangostin (24 μM /L) (48 μM /L) (12 μM /L)	↑ Inhibition of formation of biofilm biomass ↑Bactericidal activity ↑Inhibit initial adherence ↑Biological activity	[161]
*S. mutans*	*-*	Nano α-Mangostin (6.25 μM/L)	↓Biofilm biomass (49.1%) ↑Downregulate the expressions of *gtfB* and *gtfC* genes involved in biofilm formation ↑Membrane permeabilization activity was increased in a time-dependent manner a	[162]
*S. mutans*	*-*	MIC and MBC for the strain ATCC 25175 (0.625 µg/ml) for 90 m MIC and MBC for the strain KPSK (0.625 and 1.25 µg/ml) for 60 m	↑Antibacterial activity ↑Control and prevents dental plaque	[163]
*S. mutans* *P. gingivalis*	*-*	Mangostin extract (100%, 50 %, 25 %, 12.5 %, and 6.25%) 6 h for S. mutans and 24 h for P. gingivalis	↑Inhibit the bacteria growth in biofilms	[164]
*S. mutans*, oralis A. neaslundii	*-*	α-Mangostin (150 μM, twice-daily) for 60 s	↓Aggregation of bacteria Disrupts single-species biofilm formation ↓The glycosyltransferases of GtfB and GtfC^a^ ↑Oral hygiene	[165]
*S. mutans*	*-*	α-Mangostin(150 μM, twice-daily) for 60 s	↓Biomass accumulation ↓EX. insoluble and IN; iodophilic PLS ↑Remove the plaque from the sHA surface ↓Activation of enzymes glucan synthesis, acid production ↓Glucosyltransferases B,C ↓PTS system ↓F_1_F_0_-ATPase ↓Expression of manL^b^ ↓Expression of atpD^c^*, *gtfC genes ↓Development and structural integrity of biofilms bacteria ↓Exopolysaccharide synthesis and acidogenicity	[166]
*S. aureus*	*-*	α-Mangostin (48 μM /L) → (36 μM /L) →	↓Biofilm formation ↑Membrane damage (50% of cell lysis) on human RBCs	[167]
*S. aureus, B.subtilis, * *L. fermentum, S. enterica, * *E. coli, P. aeruginosa*	*-*	α-Mangostin (4-256 μM /L)	↓Vero cell toxicity ↑Antioxidant and antibacterial activities	[168]
*E. faecalis, S. aureus*	*-*	Water-soluble derived of Mangostin (0.115%)	↑ Antibacterial activity in canal root	[169]
*P. gingivalis, T. forsythia, * *A. actinomycetemcomitans*	*-*	Mangostin pericarp extraction for 30 minutes (MIC: 20 µg/ml, MBC: 40 µg/ml) (MIC: 10 µg/ml, MBC: 20 µg/ml) (MIC, MBC: >640 µg/ml)	↑ Antibacterial activity in periodontal pathogens	[170]
*S. saprophyticus*	*-*	α-Mangostin/oxacillin complex (MIC: 8 and 128 μg/ml) for 1-24 h	↑Inhibit β-lactamase in a dose-dependent manner ↑Peptidoglycan and CM damage and DNA leakage ↑CM permeability ↑ Antibacterial activity	[171]
*S. mutans*	*-*	α-Mangostin/QCD-g-CS complex (5% loaded → MIC: 0.6, IC_50_:1.04 mg/m) (15% loaded → MIC: 1.25, IC_50_:0.41 mg/m) (75% loaded → MIC: 10, IC_50_:0.22 mg/m) Depended on incubation time (1-12 h)	↑Anti-inflammatory activity: ↓TNF-α ↑ Antibacterial activity	[172]
*S. mutans*	*-*	Mangostin pericarps extraction MIC, MBC: 0.625 µg/ml (strain ATCC) MIC: 0.625, MBC: 1.25 µg/ml (strain KPSK) for 90 m	↑ Antibacterial and bactericidal activity	[173]
*H. pylori*	*In vivo*	β-Mangostin (5, 10, 20 mg/Kg)	↓The ulcer area formation, the submucosal edema, and the leukocytes infiltration ↑Gastric homogenate content of PGE2 GSH, SOD, catalase, and nonprotein sulfhydryl compounds ↓MDA, LPOs, Bax expression ↑HSP70 expression ↑Anti-H. pylori activity	[174]
	*In vitro*	α-Mangostin (MIC: 24 μg/ml)	↑Anti-bacterial activity	[175]
*S. aureus, B. anthracis*	*-*	α-Mangostin (MIC: 0.025 mg/mL, 0.013 mg/mL)	↑ Antibacterial activity ↓LPOs ↑Antioxidant	[176]
		β-Mangostin (for both strain MIN: >3 mg/mL)		
*E. coli, * *S. aureus,* *S. epidermidis,*	*-*	Mangostin extract (66 μg/ml at 48 h)	↑Growth inhibition of bacteria	[177]

**Abbreviations:** A. neaslundii;* Actinomyces naeslundii, B. subtilis; Bacillius subtilis, C. albicans; Candidia albicans,* CM; cytoplasmic membrane, CSH; Cell surface hydrophobicity, E. faecalis; Enterococcus faecalis,*E. faecalis; Enterococcus faecalis*, EPS; exopolysaccharides, EX; extracellular, *F. columnare; Flavobacterium columnare*, FIC; fractional inhibitory concentration, GMC; chloroform extract of Mangostin, GME; ethyl acetate extract of Mangostin, *H. pylori*;* Helicobacter pylori*, HC_50_;Hemolytic property, IN; intracellular*,* *L biflexa; Leptospira biflexa L. infantum*;* Leishmania infantum, L. rhamnosus*;* Lacticaseibacillus rhamnosus*, LS02; Semisynthetic γ-Mangostin derivative, *M. tuberculosis*;* Mycobacterium tuberculosis*, MBC; minimal bactericidal concentration, MDA; malondialdehyde, MIC; minimal inhibitory concentration, *P.* acne;* Propionibacterium *acne*, P. aeruginosa*;* Pseudomonas aeruginosa, P. falciparum*;* Plasmodium falciparum*, PLS; polysaccharides, PTS; phosphotransferase, PTS; phosphotransferase, S. aureus; *Staphylococcus aureus, S. enterica; Salmonella enterica, S. iniae*;* Streptococcus iniae,* S. mutans; Streptococcus mutans, sHA; saliva-coated hydroxyapatite, SOD; superoxide dismutase, *T. brucei; Trypanosoma brucei, T. cruzi*; *Trypanosoma cruzi.*

a: Key enzymes for biofilm formation, b: Encoding a key component of the mannose PTS, c: Encoding a key component of F-ATPase.

**Table 3:** Effects of Mangosteen and its xanthones against LPS and viral-induced toxicities.

**Toxin**	**Model**	**Constituent**	**Results**	**References**
LPS	*In vivo*	α-Mangostin (20, 40, 80 μM/L) for 24 h	↑Autophagy, IL-10 ↓NLRP3 inflammasome activation Improved phagocytosis and killing of macrophages ↑M2 macrophages numbers ↓TNF-α, IFN-γ, ALT, AST, Cr, IL-1β	[178]
	1.* In vitro* 2.* In vivo*	α-Mangostin (100-500 nM) in murine microglial cell line (50 mg/kg/day)orally in mice for 14 days	↓iNOS in microglia ↓TNF-α and IL-6 ↓Microglial migration and phagocytosis ↓Microglia-mediated neuronal dendritic damage ↑TLR4 expression, TAK1, and NF-κB activation ↑Anti-neuroinflammatory, neuroprotective, and memory-improving effects	[179]
LPS	*In vivo*	Mangosteen extract (50 mg/kg) and α-Mangostin (20 mg/kg) for 16 days, on schizophrenia-related bio-behavioral alterations in a maternal immune-activation rat model	↓Depressive-like behavior ↓Cortical LPOs ↓TNF-α and IL-6	[180]
	*In vivo*	α-Mangostin (40 mg/kg) for 14 days in mice	↓COX2, TSPO ↓IL-6 protein ↓Iba-1 protein in the hippocampus	[181]
	Molecular docking *In vivo*	β-Mangostin	↓NO, PGE2 production ↓TNF-α, IL-6, IL-1β cytokines Repressing translocation of NF-kB into the nucleus ↓NF-kB ↓Total leukocyte migration (neutrophils)	[182]
	*In vitro*	α-, γ-Mangostin (3 μM/L) for 24 h	↓TNF-α, IL-6, IL-1β cytokines, monocyte chemoattractant protein-1, Toll-like receptor-2 ↓MAPK, JNK1,p38, c-Jun, AP-1 activity ↓IκBα Degradation, NF-κB ↑Insulin*-*stimulated glucose uptake*,* PPARγ, adiponectin gene expression	[183]
	*In vitro*	Mangosteen peel extraction (5, 10, 20 µg/ml) α-Mangostin (25, 50, 75 µg/ml) γ-Mangostin (25, 50, 75 µg/ml)	↓COX-2, IL-1β, IL-6, and NO ↑Anti-inflammatory	[184]
	*In vitro*	α-Mangostin-NLC (62.5 μg/mL)	↑Anti-inflammatory ↑Anti-proliferative activity toward spermatogonium cells ↑Apoptosis ↓Accumulation of nitrites, TNF-α	[185]
	*In vitro*	α-Mangostin (10 mg/kg) subcutaneous injections for 7 days	↑Inhibit RANKL-induced osteoclastogenesis and osteoclast functions ↓Osteoclast-related gene expression ↓NF‑κB and MAPK signaling pathways	[186]
DENV	*In vitro*	α-Mangostin (5, 10, 20 µM) for 48-h	↓virus replication ↓TNF-α, IFN-γ ↑Anti-antiviral and anti-inflammatory effects on DENV	[187]
DENV	*In vitro*	α-Mangostin (20 μM)	↓DENV infection ↓IL-6 and TNF-α ↓Chemokine (RANTES, MIP-1β, and IP-10) transcription	[188]
DENV	*Ex vivo*	α-Mangostin (20 and 25 μM)	↓DENV production ↓Infection of immature moDCs TNF-α, CCL4, 5, CXCL10, IL-6, 10 , IL1β, and IFN-α	[189]
HCV	*In vitro*	α-Mangostin (6.3 µM) γ-Mangostin (2.7 µM) for 72 h	↓HCV replication ↓NS5A and ROS ↓HCV RNA	[190]

**Abbreviations:** Creatinine: Cr, aspartate transaminase: AST, serum alanine aminotransferase: ALT, glutathione: GSH, superoxide dismutase: SOD, catalase: CAT, toll-like receptor 4: TLR4, heme oxygenase-1: HO-1, 18 kDa translocator protein: TSPO, cyclooxygenase: COX, loaded nanostructured lipid carrier: NLC, receptor activator of nuclear factor-κB (NF-κB) ligand: RANKL, dengue virus: DENV, Hepatitis C virus: HCV, Macrophage inflammatory protein: MIP, Regulated upon Activation, Normal T Cell Expressed and Presumably Secreted: RANTES, monocyte-derived dendritic cells: moDCs, mitogen-activated protein kinase: MAPK, c-Jun NH2-terminal kinase: JNK1, activator protein-1: AP-1, peroxisome proliferator-activated receptor gamma: PPARγ. NS5A is a viral and RNA-dependent protein in the Hepatitis C virus with a crucial functional role in viral replication.

**Table 4:** Evaluation of Mangosteen and its xanthones on parasites and fungal-induced toxicities.

**Toxin**		**Model**	**Constituents**	**Results**	**References**
Parasite	*P. falciparum*	*In vitro* *In-silico*	Mangostin (33 µM)	↑More vigorous free radical scavenging activity against DPPH, a lipid-soluble free radical ↑Sensitivity of the parasite ↑Ability to interfere with hemozoin formation or the intravascular accumulation of CQ Pharmacophore to develop new CQ resistance reversing agents	[191]
		*In vitro*	α-Mangostin (10 μg/ml) for 24 h	↑Inhibition of the growth of malaria parasite ↑Inhibition of stadium development and globin accumulation	[192]
		*In vitro*	Prenylated xanthones of mangosteen	↑The anti-plasmodial activity	[193]
		*In vitro* *In vivo*	α-Mangostin (IC_50_: 0.2 ± 0.01 μM) (100 mg/kg) i. p, twice a day for 7 days	↑The anti-plasmodial activity	[194]
	*C. elegans*	*In vitro*	α-Mangostin (IC_50_: 3.8 ± 0.5 μM)	↑Cytotoxic effect of the drug, even by 4- and 25-fold ↑The anti-nematode activity	[195]
		*In vivo*	α-Mangostin (IC_50_: 18.74)	↑The anti-nematode activity	[196]
	*A. triangularis*	*In vivo*	α-Mangostin (trophozoite, MIC: 0.25, 0.5 mg/mL) (Cysts, MIC: 4, 1 mg/mL) for 72 h	↑Damage to the cell membrane and irregular cell shapes ↓Acanthamoeba infection	[197]
		*In vitro*	Mangosteen extract (MIC: 0.25–1 mg/mL)	↓Virulence factor associated with the adhesion of A. triangularis	[198]
Fungi	*C. elegans*	-	α-Mangostin (0.1, 1, 10 μM) 96 h	↑Fat accumulation in worm ↑Movement speed ↑Thermos-tolerance in C. elegans	[199]
	*C. albicans*	*In vitro*	α-Mangostin (MIC: 1,000, MFC: 2,000 μg/ml)	↑Fungicide activity	[200]
	*C. albicans*	*In vitro*	Mangostin extract (500, 1000 ppm) for 60 min	↓The viability of C. albicans ↑Promising adjuncts in oral health product	[201]
	C. gloeosporioides	*In vitro*	α-Mangostin (MIC: 100 μg/mL)	↑Inhibitory effect on spore germination ↑Accumulation of dense ↓Quantity and shape of the swelling of mitochondria in the mycelium cell bodies	[80]
	3-NP	-	α-Mangostin	↑Scavenge directly several ROS ↑Neuroprotective effect against 3-NP	[202]
Arthropod	*L. decemlineata*	*In vitro*	α-Mangostin: LC_50_: 63.66 (13.29-279.79, 8.37 (2.67-20.27 4.09 (0.89-11.81) μM For 7-, 14- and 23-days treatment	↑Larvicidal activity ↑Growth inhibitory ↑Anti-feeding activity in adult beetle	[203]
	*Aedes aegypti*	*In vitro*	Mangostin pericarp extract (LC_50_: 4.84 mg/L and 6.19 mg/L LC_90_: 14.55 mg/L and 28.71 mg/L)	↑LarvIcidal activity	[204]
	Mosquitoes	*In vitro*	α-Mangostin: 0.84 to 2.90 ppm	↑P450 and GSH activity in larvae ↑Suppressed esterase activity	[205]

**Abbreviations:** *A. triangularis: Acanthamoeba triangularis*, *C. elegans: Caenorhabditis elegans*, *C. glabrata: Candida glabrata*, C. gloeosporioides: Colletotrichum gloeosporioides, CQ: chloroquine, DPPH: 2,2-Diphenyl-1-picrylhydrazyl, i.p: intraperitoneal, *L. decemlineata: Leptinotarsa decemlineata*, MFC: minimum fungicidal concentration, MIC: minimum inhibitory concentration, NP: 3-nitropropionic acid, *P. falciparum: plasmodium falciparum*.

**Table 5:** Evaluation of Mangosteen and its xanthones on various toxic compounds.

**Agent**	**Model**			**Constituents**	**Results**	**References**
H_2_O_2_	*In vitro*			γ-Mangostin (3~10 μM)	↓DNA fragmentation ↓Activation of caspases 3, 9 ↑Antiapoptotic action ↓ROS production	[206]
	*In vitro* Molecular-docking			α-Mangostin (1 μM) for 3 h	↓Oxidative stress-induced cell death in neuronal cells ↓BAX protein, caspase-3/7 activation ↑Apoptotic BCL-2 protein ↓ROS production ↑Antioxidant enzymes (CAT and SOD2) ↑Sirtuin family and the FOXO3a transcription factor Directly bound to the active site of SIRT1	[207]
	*In vitro*			Water and 50% ethanol extracts of Mangostin (IC_50_: 34.98 ± 2.24 and 30.76 ± 1.66 μg/mL)	↑High free-radical scavenging activity ↑Neuroprotective activity	[208]
	*In vivo*			Mangostin water extract (50-500 μg/mL) for 3 h	↑Protected cells from oxidative damage ↓Lipid peroxide	[209]
	*In vivo*			Mangostin extract (100 mg/kg )	↓Escape latency ↑Learning ability	[210]
	*In vitro*			Mangostin extract: (200 μg/ml) for 3 h→ (200-800 μg/ml) for 24 h→	↓Cytotoxic effect of H_2_O_2_ ↓Intracellular ROS production ↓AChE	
Alcohol	*In vivo*			γ-Mangostin (10 mg/kg, 20 mg/kg) *H. pylori*	↑Gastroprotective effect ↑Gastric mucosal content and gastric juice pH ↑Mucosal Hsp70 protein ↓Bax proteins ↑SOD and CAT activities ↓MDA ↓Alcohol-induced oxidative stress	[211]
	*In vivo*			α-Mangostin (10 and 30 mg/kg)	↓Gastric lesions ↑Expression of Hsp70 and restores GSH ↓LPOs and COX-2 activity	[175]
TAA	*In vivo*			α-Mangostin (5 mg/kg)	↓Collagen fibers in Bowman’s capsule and the interstitium ↓Fibrosis ↓Cflar, Lamtor3, Map3k14, and Mapk8ip3	[212]
	*In vivo*			α-Mangostin	↓Decline CPS and GS immuno-reactivity from week eight to sixteen ↑Normal expression of ammonia-metabolizing enzymes	[213]
	*In vivo*			α-Mangostin (75 mg/kg) twice per week, ip, for 30 and 60 days	↓Hepatocellular injuries, periportal and pericentral fibrosis ↑Preservation of the hepatic microvascular pattern ↓Sinusoidal patterns alteration with signs of terminal HPV remodeling	[214]
	*In vivo*			α-Mangostin (5 mg/kg) for 8 weeks	↓TGF-β1, α-SMA and TIMP-1 ↓Fibrosis	[215]
	*In vivo*			α-Mangostin (100 mg/kg) 3 times per week for 4 weeks	↓Fibrotic nodules ↓AST and ALT ↓p53 protein expression in the liver	[216]
Alloxan	*In vivo*			α-Mangostin (5, 10, 20 mg/kg) for 21 days	↓FBS ↑Insulin plasma ↑Improvement of the islet of Langerhans ↑Protective effect on islet Langerhans cells from oxidative stress	[217]
	*In vivo*			α-Mangostin (30 mg/kg)	↓Blood glucose	[218]
CCl_4_		-	Jungle Mangosteen extract (500 mg/kg) for 24 h		↓Hepatotoxicity ↓ALP, ALT and AST	[219]
		*In vitro*	γ-Mangostins (5 μM) for 48 h		↓Collagen I and α-SMA ↓NADPH oxidase activity *via *SIRT3 stimulation ↓Intracellular oxidative stress, histone deacetylase 1 ↓Acetylation and cytoplasmic shuttling of HMGB1	[220]
		*In vivo*	γ-Mangostins (5 and 10 mg/kg) for 30 days		↑The expression of SIRT3 ↓HMGB1 ↓Accumulation of collagen I and α-SMA in liver	
DMH		*In vivo*	α-Mangostin (0.02% and 0.05%) for 5 weeks		↓Induction/development of ACF ↓Dysplastic foci, BCAC, PCNA	[221]
DSS		*In vivo*	Mangosteen extract (40, 200, and 1000 mg/kg) α-Mangostin (30 mg/kg) for 7 days		↓UC disease activity index ↓Histological damage Protective effect on colon shortening and spleen and kidney enlargement ↓Mast cell infiltration in the colon ↓Myeloperoxidase activity ↓ROS, NO, and MDA production ↑SOD, GSH	[222]
Rotenone		*In vivo*	α-Mangostin (25 mg/kg) for 48 h		↓Motor deficits, ↑TH intensity in striatum and SNc ↓Reduction of neurons in SNc ↓Reduction of neurons in the striatum and motor cortex ↓MDA ↑SOD, GSH	[223]
PPA		*In vivo*	α-Mangostin (100, 200 mg/kg) for 32 days		↓Overactivation of the ERK signaling Preserve neurochemical alterations and autism-like behavioral ↓Body weight loss ↑Long-term memory improvement ↑Muscle coordination ↓Immobility ↓Increased ERK level ↓Caspase-3, Bax, and Bcl-2 Levels ↑Serotonin, dopamine ↓Glutamate ↓TNF-α, IL-1β, AchE, LDH, MDA, and Nitrite ↑SOD, GSH	[224]
CoCl_2_		*In vitro*	α-Mangostin (0.06 and 0.3 mM)		↑Cell viability ↓ROS production, MDA, cellular apoptosis ↑SOD ↓Cleavage of caspase‑9, 3 and BAX ↑Bcl‑2 Improve hypoxic-induced cardiac injury	[225]
FeSO_4_ 3-NP Quinolinate		*In vitro*	α-Mangostin (10–500 µM)		↓Lipoperoxidative action ↓Decreased reductant capacity of mitochondria in synaptosomal fractions	[226]
FeSO_4_ 3-NP		*In vitro*	α-Mangostin: FeSO_4_: (25, and 50 μM) 3-NP: (10, 25, and 50 μM)		↑GPx activity ↑GSH	[227]
IAA		*In vitro*	α-Mangostin (8-14 μM)		↑Prevent about 60% of the IAA-induced cell death ↓The ROS production ↑Induce HO-1 expression	[228]
PMA		*In vitro*	α-Mangostin (5μM)		↓PMA-induced abilities of the adhesion, invasion, and migration ↓Activation of alphavbeta3 integrin, FAK, and ERK1/2 by FAK siRNA ↓Degradation of IkappaBalpha, the NF-kappaB ↓MMP-2, -9 gene expressions	[229]

**Abbreviations:** ACF: aberrant crypt foci, AChE: acetylcholinesterase, BCAC: β-catenin accumulated crypts, CCl_4_: carbon tetrachloride, Cflar: CASP8 and FADD-like apoptosis regulator, CPS: carbamyol phosphate synthetase, DMH: 1,2-dimethylhydrazine, DSS: dextran sulfate sodium, ERK ½: extracellular signal-regulated kinase1/2, FAK siRNA: FAK small interfering RNA, FAK: focal adhesion kinase, FBS: fasting blood glucose, FeSO_4_: ferrous sulfate, GPx: glutathione peroxidase, GS: glutamine synthetase, H_2_O_2_: Hydrogen Peroxide, HMGB1: high mobility group box 1, HPV: hepatic portal venule, IAA: iodoacetate, IkappaBalpha: inhibitor of kappaBalpha, ip: intraperitoneally, Lamtor3: regulator complex protein LAMTOR3, Map3k14: mitogen-activated protein kinase kinase kinase 14, Mapk8ip3: C-Jun-amino-terminal kinase-interacting protein 3, MMP: matrix metalloproteinase, NF-kappaB: nuclear levels of nuclear factor kappa B, 3-NP: 3-nitropropionate, PCNA: proliferating cell nuclear antigen, PMA: phorbol 12-myristate 13-acetate, PPA: Propionic Acid, SIRT3: sirtuin 3, SNc: substantia nigra par compacta, SOD: superoxide dismutase, TAA: thioacetamide, TH: tyrosine hydroxylase, TIMP-1: metalloproteinase 1,UC: Ulcerative colitis, α-SMA: alpha-smooth muscle actin.

**Table 6:** Evaluation of Mangosteen and its xanthones on chemical-induced toxicities.

**Chemical**	**Model**	**Constituents**	**Results**	**References**
Cisplatin	*In vitro*	α-Mangostin (5-40 μM) for 24 h	↓Cell death ↓MDA ↑GSH Restored PI3K/AKT ↓JNK ↓ROS-mediated caspase pathway	[230]
	*In vivo* (rat)	α-Mangostin (12.5 mg/kg/day, i.g.) for 10 days (7 days before and 3 days after CDDP injection)	↓Renal dysfunction ↓Structural damage in the kidney ↓Oxidative/nitrosative stress ↓CAT expression ↓mRNA levels of TNF-α and TGF-β expression	[102]
	*In vitro*	α-Mangostin (1-5 μM) for 24 h	↓Cell death and CDDP-induced damage in proximal tubule cells ↓Decrease cell respiratory states and respiration associated with OXPHOS ↑VDAC and mitochondrial complex subunits Improve mitochondrial morphology alteration, mitochondrial mass, and mitochondrial biogenesis.	[231]
	*In vitro*	α-Mangostin (5 μM) for 24 h	↓Apoptotic cell death ↓ROS ↓GSH depletion ↓p53 expression	[232]
Olanzapine	*In vivo* (rat)	α-Mangostin (10, 20, 40 mg/kg/day, IP) for 14 days	↓Weight gain, food intake, systolic blood pressure, triglycerides, LDL, blood sugar, leptin, and MDA ↑GSH, AMPK, and P-AMPK in liver tissue	[233]
STZ	*In vivo* (mice)	γ-Mangostin (0.5, 1, 2 mg/kg) for 14 days	↓FBS, cholesterol, SGOT, SGPT ↑Ameliorate the damaged hepatocytes	[112]
	*In vivo* (mice)	Mangosteen extract (50, 100, 200 mg/kg) for 14 days	↓Plasma creatinine level ↑Ameliorate the proximal renal tubule damage ↑Antidiabetic and antioxidant activities	[234]
	*In vivo* (mice)	α-Mangostin (2, 4, 8 mg/kg) for 14 days	↑Hypoglycemic and hypolipidemic activities ↑Ameliorate the damaged islets of Langerhans	[235]
STZ	*In vivo* (rat)	α-Mangostin (25, 50 and 100 mg/kg, p.o.)	↓Blood glucose enhanced body weight ↑Utilization of glucose by different organs ↑Plasma insulin, hemoglobin, hexokinase, HDL, total protein, SOD, CAT, GSH ↓Glycated hemoglobin, fructose-1-6-biphosphatase, glucose-6-phosphatase, TC, TG, LDL, VLDL, CRE, BUN, SGOT, SGPT, ALP, and LPO ↓Inflamed blood vessels in diabetic kidney ↑Improved β cells, islets, acini, and producing necrosis ↑Improved damaged hepatocytes as well as central vein	[236]
	*In vivo* (rat)	α-Mangostin (100 and 200 mg/kg) for 8 weeks	↓MDA ↑GSH, SOD in the heart, liver, and kidney tissues ↑Skeletal glucose transporter type 4 expression	[237]
	*In vitro*	α-Mangostin (1-10 μM)	↑Insulin secretion in INS-1 cells by activating insulin receptor (IR) and pancreatic and duodenal homeobox 1 (Pdx1) ↑Phosphorylation of PI3K, Akt, and ERK signaling cascades ↓IRS-1 (Ser1101) ↑Restore INS-1 cell viability ↓ROS ↓Phosphorylation of P38, JNK, and cleavage of caspase-3	[238]
	*In vivo* (rat)	α-Mangostin (25 and 50 mg/kg) for 55 days	↑Sperm count, motile sperms, viable sperms, and hypo-osmotic swelling tail coiled sperms ↑Serum testosterone levels and testicular 3β and 17 β-hydroxysteroid dehydrogenase levels ↓Sperm malformations ↑SOD, catalase, GPx levels ↓LPOs production	[239]
	*In vivo* (rat)	Mangosteen extract (50, 100, and 200 mg/kg) for 28 days	↓Blood glucose level ↑Body weight ↓TG, TC, LDL, VLDL, SGOT, SGPT, urea, and creatinine ↑HDL and TP	[240]
DOX	*In vivo* (mice)	Mangosteen (200 mg/kg)	↓TNFα ↓Carbonyl, nitrotyrosine, and 4HNE in brain tissue ↓Pro-apoptotic proteins p53 and Bax and the anti-apoptotic protein Bcl-xL ↓Caspase-3 activity and TUNEL-positive cells ↓ROS	[241]
	*In vitro*	α-Mangostin (0, 3.75, 7.5, and 15 μM) for 24 h	↑Cell viability ↓Apoptosis, Bax, cleaved PARP-1, cleaved caspase-3 protein expression ↑Bcl-2 protein ↓Intracellular ROS and extracellular H2O2 generation ↓Abnormal enzyme activities of SOD, CAT, and GSH were regulated by reducing PI3K, AKT, PGC-1α, and STRT-3 signaling in ARPE-19 cells.	[242]
	*In vivo*	α-Mangostin (20 mg/kg)	Improved retinal deformation ↑Inhibiting the expression of cleaved caspase-3 →the ↑Thickness of both the outer and inner nuclear layers	
Iodixanol	*In vivo*	α-Mangostin (2.5 and 5 μM) for 3 h	↑Viability cells ↓Phosphorylation of p38, ERK, and cleavage of caspase-3 Inhibition of MAPKs and caspase activation	[243]
Scopolamine	*In vivo* (mice)	Mangosteen extract (100 mg/kg) for 14 days	↓Escape latency time ↓ROS levels and caspase-3 activity ↑KPNB1 level	[210]
Scopolamine	*In vivo* (mice)	γ-Mangostins (10 and 30 mg/kg)	Improved memory impairment ↓time latency	[206]
Acetaminophen	*In vivo* (mice)	α-Mangostin (100 and 200 mg/kg) for 7 days	↑GSH ↓MDA, TNF-α, IL-1β ↓Expression of autophagy-related microtubule-associated protein LC3 and BCL2/adenovirus E1B protein-interacting protein 3 ↓Apoptotic signaling pathways ↑Expression of Bcl-2 ↓Bax and cleaved caspase 3 proteins ↓p62 level, p-mTOR, p-AKT, and LC3 II /LC3 I ratio in autophagy signaling pathways	[244]
ISO	*In vivo* (rat)	α-Mangostin (200 mg/kg body weight) for 8 days	↑Respiratory chain enzymes: NADH dehydrogenase and cytochrome c oxidase ↑Tricarboxylic acid cycle enzymes: IDH, SDH, MDH, and α-KGDH ↑Mitochondrial antioxidants (GPx, GST, SOD, CAT, and GSH) ↑Mitochondrial cytochromes (b, c, c1, and aa3), and ATP level ↓Mitochondrial LPOs ↑Restored normal mitochondrial function Improve eNOS expression and NO level	[245]
	*In vivo* (rat)	α-Mangostin (200 mg/kg body weight) for 8 days	↓Cardiac lysosomal hydrolases: *β*-d-glucuronidase, *β*-d-galactosidase, *β*-d-*N*-acetylglucosaminidase, acid phosphatase and cathepsin-D ↓Na, Ca in cardiac ↓K paralleled by abnormal activities of membrane-bound phosphatases: Na^+^–K^+^ ATPase, Ca^2+^ ATPase and Mg^2+^ ATPase ↓TNF-α and COX-2 expressions	[246]
Bafilomycin-A1 CQ Rapamycin	*In vitro*	β-Mangostin (2.5, 5, 10 μM)	↑Tyrosinase degradation ↓p62, melanosome proteins (tyrosinase and PMEL) ↑Depigmentation effect ↓Melanogenesis *via *the autophagy pathway ↑Degrading melanosomes	[247]
Paclitaxel	*In vitro*	α-Mangostin (0.1, 0.15, 0.25, 0.5, 1 μM) for 24 h	↑p53 activation in cell cycle arrest for low-level stressors ↓Binding of p53 to MDM2 ↑Protective effect on the health cells ↓The side effects of paclitaxel	[248]
		α-Mangostin (10 μM)	↑p53 activation in cell cycle arrest for low-level stressors ↓Binding of p53 to MDM2 ↑Protective effect on the health cells ↓The side effects of paclitaxel	[249]
Bleomycin	*In vivo* (mice)	α-Mangostin (10 mg/kg/day) for 14 days	↓The body weight loss and survival rate reduction ↓Lung coefficient ↑Ameliorated pulmonary histopathological changes ↓Progression of fibrosis ↓α-SMA and collagen I expression at mRNA and protein levels ↓MMP-9 and TIMP-1 expression ↓TGF-β1/Smad2/3 signaling pathway, NOX4 expression ↑AMPK activation in the lungs	[250]
	*In vitro*	α-Mangostin (1-50 nM) for 48 h	↑p-AMPK/AMPK ↓The protein expression level of α-SMA, Col I, NOX4	

**Abbreviations:** AMPK: AMP-activated protein kinase, AKT: protein kinase B, ATP: adenosine triphosphate, CAT: catalase, CQ: chloroquine, DOX: doxorubicin, ERK: extracellular signal regulated kinase, FBS: fasting blood glucose, GSH: glutathione, HDL: high density lipoprotein, 4-HNE: 4-hydroxy-2′-nonenal, IDH: isocitrate dehydrogenase, IRS-1: phosphorylation of insulin receptor substrate, ISO: isoproterenol, JNK: c-Jun N-terminal kinase, JNK: JUN N-terminal kinase pathway, KPNB1: Karyopherin β1, LC3: light chain 3, LDL: low density lipoprotein, MDA: malondialdehyde, MDH: malate dehydrogenase, MMP: Matrix metalloproteinase, NOX4: nicotinamide adenine dinucleotide phosphate oxidase-4, OXPHOS: oxidative phosphorylation, PI3K: phosphatididylinositol 3-kinase, ROS: reactive oxygen species, SGOT: Serum glutamic oxaloacetic transaminase, SGPT: Serum Glutamic Pyruvic Transaminase, SOD: superoxide dismutase, STZ: Streptozotocin, Succinate dehydrogenase, TC: total cholesterol, TG: triglycerides, TIMP: tissue inhibitor of metalloproteinase, TNF-α: tumor necrosis factor alpha, TP: total protein, TGF-β: transforming growth factor beta, VDAC: voltage dependence anion channel, VLDL: very low density lipoprotein, α-KGDH: alpha-ketoglutarate dehydrogenase, α-SMA: α-smooth muscle actin, Carbonyl: markers of protein oxidation, 3-NT: a marker of protein nitration, 4HNE: a LPOs product, α-SMA: marker protein of myofibroblast.

## LIST OF ABBREVIATIONS

A. bisexualis: Achlya Bisexualis
A. invadans: Aphanomyces Invadans
ABTS: 2′-azino-bis-3-ethyl benzthiazoline-6-sulphonic Acid
ALT: Serum Alanine Aminotransferase
AMPK: AMP-activated Protein Kinase
AST: Aspartate Transaminase
AVP: Avian Pox Virus
BPH: Brown Planthopper
*C. albicans*: *Candida albicans*
*C. elegans*: *Caenorhabditis elegans*
CAT: Catalase
*CD3*: Cluster of Differentiation 3
3D7: Chloroquine-sensitive Plasmodium Falciparum
COX: Cyclooxygenase
Cr: Creatinine
DPPH: 2,2-Diphenyl-1-picrylhydrazyl
*G. mangostana*: *Garcinia mangostana*
GSH: Glutathione
H_2_O_2_: Hydrogen Peroxide
Hap: Hydroxyapatite
HIV: Human Immunodeficiency Virus
HO-1: Heme Oxygenase-1
IC50: Inhibiting Concentration 50
IL: Interleukin
INF-γ: Interferon-gamma
IR: Ionizing Radiation
K1: Chloroquine-resistant Plasmodium Falciparum
LPOs: Lipid Peroxidation
MAPK: Mitogen-activated Protein Kinase
MC3T3-E1: Calvaria-derived Pre-osteoblastic
NF-κB: Nuclear Factor-κB
Nrf2: Nuclear Factor Erythroid 2-related Factor 2
O. viverrini: Opisthorchis viverrini
P. falciparum: Plasmodium falciparum
PG: Prostaglandin
ROS: Reactive Oxygen Species
*S. diclina* H3: *Saprolegnia diclina* H3
SOD: Superoxide Dismutase
TLR4: Toll-like Receptor 4
TNF: Tumor Necrosis Factor
α-Syn: α-synuclein
